# Progressing beyond SLMTA: Are internal audits and corrective action the key drivers of quality improvement?

**DOI:** 10.4102/ajlm.v3i2.222

**Published:** 2014-11-03

**Authors:** Robert N. Maina, Doris M. Mengo, Abdikher D. Mohamud, Susan M. Ochieng, Sammy K. Milgo, Connie J. Sexton, Sikhulile Moyo, Elizabeth T. Luman

**Affiliations:** 1 Kenya Accreditation Service (KENAS), Kenya; 2 US Centers for Disease Control and Prevention, Atlanta, United States; 3 Botswana-Harvard AIDS Institute Partnership, Princes Marina Hospital, Botswana

## Abstract

**Background:**

Kenya has implemented the Strengthening Laboratory Management Toward Accreditation (SLMTA) programme to facilitate quality improvement in medical laboratories and to support national accreditation goals. Continuous quality improvement after SLMTA completion is needed to ensure sustainability and continue progress toward accreditation.

**Methods:**

Audits were conducted by qualified, independent auditors to assess the performance of five enrolled laboratories using the Stepwise Laboratory Quality Improvement Process Towards Accreditation (SLIPTA) checklist. End-of-programme (exit) and one year post-programme (surveillance) audits were compared for overall score, star level (from zero to five, based on scores) and scores for each of the 12 Quality System Essential (QSE) areas that make up the SLIPTA checklist.

**Results:**

All laboratories improved from exit to surveillance audit (median improvement 38 percentage points, range 5–45 percentage points). Two laboratories improved from zero to one star, two improved from zero to three stars and one laboratory improved from three to four stars. The lowest median QSE scores at exit were: internal audit; corrective action; and occurrence management and process improvement (< 20%). Each of the 12 QSEs improved substantially at surveillance audit, with the greatest improvement in client management and customer service, internal audit and information management (≥ 50 percentage points). The two laboratories with the greatest overall improvement focused heavily on the internal audit and corrective action QSEs.

**Conclusion:**

Whilst all laboratories improved from exit to surveillance audit, those that focused on the internal audit and corrective action QSEs improved substantially more than those that did not; internal audits and corrective actions may have acted as catalysts, leading to improvements in other QSEs. Systematic identification of core areas and best practices to address them is a critical step toward strengthening public medical laboratories.

## Introduction

Accurate, timely and affordable medical diagnosis to support patient care and management remains a challenge in developing countries. Many laboratories are ill prepared to respond to health emergencies, yet their services are critical for the detection of new pathogens and containment of disease outbreaks.^1^ Establishing a quality management system (QMS) to support accreditation is a demanding process requiring great organisational skills, motivation and huge investment that may be both overwhelming and unfeasible for public medical laboratories with limited resources. In addition to the cost of developing these systems, barriers are raised by the lack of awareness amongst healthcare workers regarding the benefits of accreditation on health services. Several publications have highlighted the advantages of a comprehensive QMS in a laboratory setting along with recognition of areas of QMS that were at risk of failing.^2,3,4,5^ Accreditation is now widely recognised as being an essential element of strengthening the QMS in public health laboratories and may also have a positive effect on other sectors of the healthcare system.^6,7^

The past few years have seen increased support for health systems in Africa through funding from initiatives such as the US President’s Emergency Plan for AIDS Relief (PEPFAR); the Global Fund to Fight AIDS, Tuberculosis and Malaria; the Global Health Initiative; and organisations such as the World Bank.^8^ Strengthening integrated laboratory services within public health laboratories, as opposed to focusing on disease-specific programmes, has been encouraged as a means of establishing a cost-effective laboratory system.^9^ The growing recognition of the importance of laboratory services has resulted in the launch of several important initiatives including the Strengthening Laboratory Management Toward Accreditation (SLMTA) programme.

SLMTA was developed by the US Centers for Disease Control and Prevention (CDC) in collaboration with the American Society for Clinical Pathology (ASCP), the Clinton Health Access Initiative and the World Health Organization’s Regional Office for Africa (WHO AFRO), with the aim of promoting immediate measurable improvement in the laboratories of developing countries.^10,11,12^

The Kenya Health Service delivers medical laboratory services through a network of 958 laboratories, of which 70% belong to the government, 20% to nongovernmental organisations and 10% to the private sector.^13^ As of the end of 2012, eight (< 1%) of the 958 laboratories in Kenya had been accredited, none of which were in the government sector.^4^ As accreditation of health facilities is a key goal of the country’s 2008–2012 Strategic Plan,^13^ Kenya’s Ministry Of Health (MOH) established a National Accreditation Steering Committee to coordinate laboratory accreditation activities. After SLMTA’s launch in July 2009, the MOH, with support from CDC’s Kenya office, adopted the programme and enrolled 53 laboratories in six cohorts between 2010 and 2013 from amongst national reference, provincial, and district-level laboratories. The MOH set a target to accredit at least five laboratories from the first cohort of 13 to the International Organization for Standardization (ISO) 15189 standard by the end of 2014. Six main partners have helped implement SLMTA in Kenya: ASCP, A Global Health Care Public Foundation (AGHPF), Management Sciences for Health (MSH), the Kenya AIDS Vaccine Initiative (KAVI), the African Field Epidemiology Network (AFENET) and the World Bank. Twenty laboratories so far have graduated from the SLMTA programme.

After completing the SLMTA programme, laboratories still had substantial quality gaps to fill before seeking accreditation. This paper reviews the performance of five laboratories from the first SLMTA cohort in Kenya, highlighting the progress made in the year after completion of the programme and identifying areas critical to implementation and sustainability of a sound QMS.

## Research methods and design

### Site selection

Five laboratories from the first SLMTA cohort in Kenya were selected for this evaluation based on two primary factors: they had the same group of mentors and mentorship schedules after the exit audit; and all were audited during the same timeframe by the same team of auditors. For the purpose of confidentiality these laboratories are presented as A–E. Laboratory B is a private clinical research laboratory, whilst the other four are government-run provincial-level laboratories with significant automation in haematology and flow cytometry but only semi-automated equipment in clinical chemistry.

### SLMTA training

The SLMTA training for the first cohort took place in 2010 and consisted of three workshops of four days each. Module one and cross-cutting activities were taught in workshop one, modules two to six were taught in workshop two and modules seven to 10 were taught in workshop three. The trainers were SLMTA Training-of-Trainers (TOT) graduates employed by CDC’s Kenya office. Two staff members from each of the five laboratories participated in the workshops. These participants were quality managers and laboratory managers, whose responsibilities included overseeing the implementation of QMS, and training and supervising the laboratory personnel. These workshops were interspersed with periods of three months, during which participants implemented improvement projects based on what was learned at the previous workshop and laboratory-specific quality gaps.

### Audits

Baseline audits were conducted in April 2010, one month before the first SLMTA workshop. Since they were conducted by non-certified auditors, their findings are not included in this analysis.

Exit audits were conducted in August 2011, eight months after the third workshop. Surveillance audits were conducted one year later, in August 2012.

Audits were conducted using WHO AFRO’s Stepwise Laboratory Quality Improvement Process Towards Accreditation (SLIPTA) checklist. The SLIPTA checklist comprises 111 questions subdivided into 12 sections that represent the quality systems essentials (QSEs) from the Clinical and Laboratory Standards Institute.^14^ For each positively answered question, points were allocated (two, three or five); partially positive answers were awarded one point. A star rating was assigned as follows: five stars (244–258 points, ≥ 95% compliance), four stars (219–243 points, 85% – 94% compliance), three stars (193–218 points, 75% – 84% compliance), two stars (167–192 points, 65% – 74% compliance) and one star (142–166 points, 55% – 64% compliance). A score of 141 points or less (< 55%) received zero stars.^15^

Both exit and surveillance audits were conducted by auditors certified by The Kenya Accreditation Service (KENAS), the sole national accreditation body in Kenya. Auditors were selected on the basis of training and technical expertise, as well as having had no previous engagement with the laboratory being audited to safeguard impartiality. All auditors had completed core training on SLIPTA as well as training on ISO requirements for quality and competency in medical laboratories (ISO 15189) and guidelines for QMS auditing (ISO 19011), had attended an annual assessor refresher course and had conducted at least one SLIPTA audit. The composition of each audit team was based on the scope of the laboratory being audited.

Methods of audit included: (1) review of documents; (2) review of records (procedures, minutes of the meetings, quality control data, corrective actions, work plans); (3) staff interviews; and (4) observation and witnessing of testing procedures. The interviews were open-ended questions used to obtain information required by the SLIPTA checklist. The laboratory management and staff received prior notification of the audit dates and auditors’ names. The auditors selected the staff and the procedure to be observed, using sampling techniques that ensured objectivity and impartiality of the audit process. This ensured that staff did not have prior knowledge of who was to be observed. Audits were conducted by two auditors per laboratory over a two-day period. Each auditor recorded their findings in their checklist before compiling the report in a consensus checklist that included the final score and the star rating for each laboratory. A summary report of all findings (both positive and negative) and a corrective action request form (for each nonconformity) were completed by the auditors on site and submitted to the laboratory management at the end of the second day of audit. A senior laboratory staff member was required to sign each form so as to acknowledge receipt of the findings. Any divergent opinions between the auditors and the laboratories were resolved on site before writing of the final report.

### Mentorship

Mentorship during SLMTA implementation was unstructured, with mentors visiting laboratories for unspecified periods of time without concrete work plans. This deficiency was partly because of a lack of training for mentors, as well as the lack of a proper mentorship programme in the country by 2010. In contrast, after the exit audit new mentors were embedded in the five facilities for two weeks per month for four months. These mentors followed comprehensive work plans in the eight weeks they were in the facilities, which covered the entire SLIPTA checklist. The mentors were consultants in QMS implementation in medical laboratories contracted by the implementing partner. Their qualifications included diploma or a bachelor’s degree in Medical Laboratory Sciences, quality management training based on ISO 15189 and the SLIPTA checklist, and experience working in an accredited laboratory or one undergoing the accreditation process.

### Data analysis

Retrospective data mining and analysis were conducted using SLIPTA audit data comprehensive reports and corrective action forms for each laboratory at the KENAS offices. We compared the exit and surveillance audit scores, evaluated performance in the individual QSEs and identified key areas of progress and challenges in implementation of a sound QMS. We also examined nonconformities at the exit and surveillance audits with a focus on identification of common issues. Any nonconformity identified at both the exit and surveillance audits in the same laboratory was classified as a recurring nonconformity. Statistical analysis was done using GraphPad Prism software Version 6.02 (GraphPad Software, La Jolla, California, USA, www.graphpad.com) and Microsoft^®^ Excel.

## Results

**FIGURE 1:**
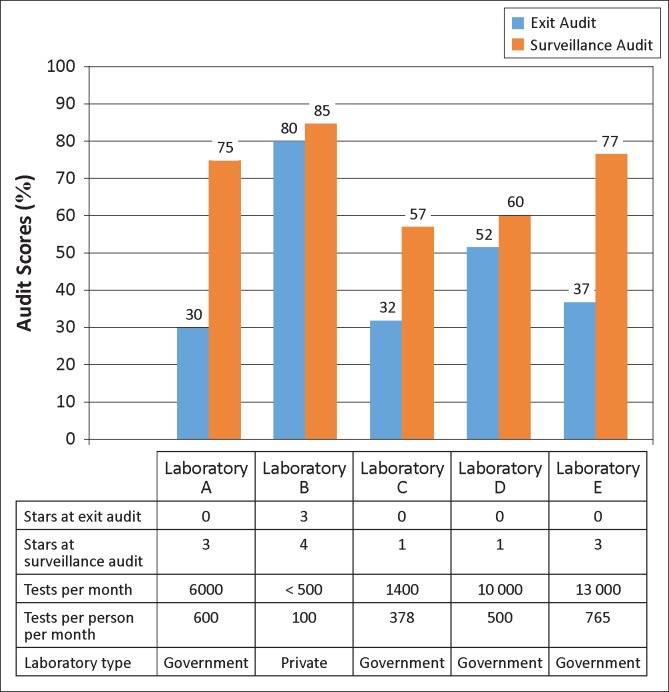
Performance of the five laboratories based on Stepwise Laboratory Quality Improvement Process Towards Accreditation (SLIPTA) checklist scores and star ratings.

Exit audit scores were below the one-star level for all laboratories except B, which scored three stars at exit. All of the laboratories increased their scores from exit to surveillance audit; two achieved one star, two achieved three stars and the laboratory that began at three stars achieved four stars (Figure 1). Median scores increased from 37% at exit to 75% at surveillance audit (improvement range 5–45 percentage points).

The two laboratories with the highest per-person workload (A and E) had the most improvement from exit to surveillance audit, whilst the laboratory with the lowest volume (B) and highest exit score had the least improvement (Figure 1).

**FIGURE 2:**
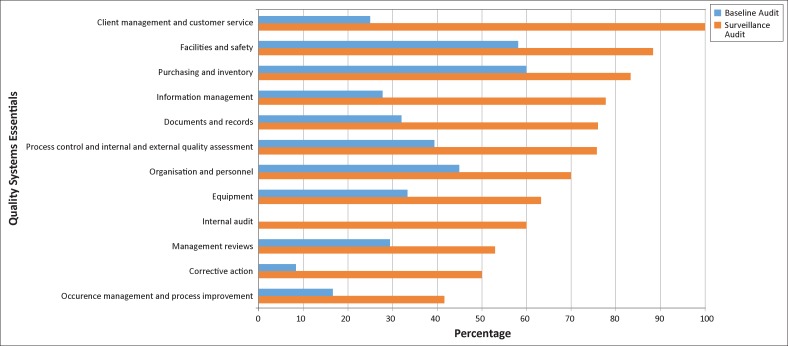
Median scores of the five laboratories in the 12 Quality System Essentials (QSEs).

The lowest median QSE scores at exit were internal audit, corrective action and occurrence management, and process improvement (all < 20%) (Figure 2). The highest medians were in purchasing and inventory, facilities and safety, and organisation and personnel (all > 45%). Each of the 12 QSEs improved substantially at the surveillance audit (range 24 – 75 percentage points), with the greatest improvement in client management and customer service, internal audit and information management (≥ 50 percentage points). The smallest improvements were in purchasing and inventory, management reviews, organisation and personnel, and occurrence management and process improvement (≤ 25 percentage points). By the surveillance audit, the areas of greatest challenge were occurrence management and process improvement, corrective action and management reviews (all ≤ 55%), whilst the strongest areas were client management and customer service, facilities and safety, purchasing and inventory, and information management (all ≥ 78%) (Figure 2).

**FIGURE 3:**
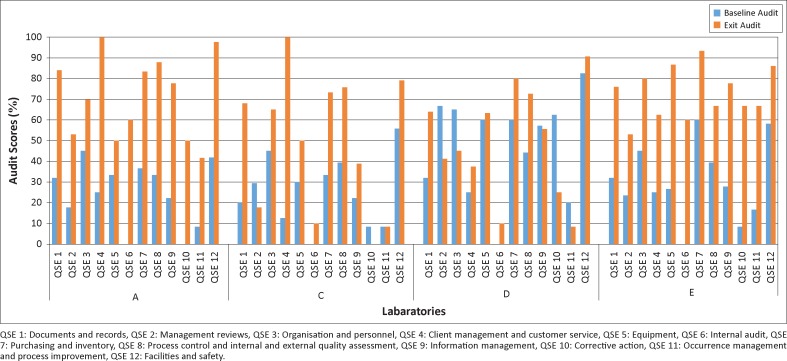
Performance of laboratories A, C, D and E in the 12 Quality System Essentials (QSEs).

In order to examine more closely the relationship between specific areas of improvement and overall success, we compared results for laboratories A and E, which started with zero stars at exit (median 34%) and reached three stars at surveillance (median 76%), to those for laboratories C and D, which also started at zero stars (median 42%) and reached one star at surveillance (median 59%). We excluded laboratory B in this sub-analysis because it was markedly smaller than the others and started with a substantially higher score at exit. Most striking were the results for the internal audit and corrective action QSEs. All four of these laboratories scored 0% in internal audit at exit. Laboratories A and E both excelled in this area post-SLMTA, increasing their score to 60% by the surveillance audit. Laboratories C and D, on the other hand put little focus on this area, scoring only 10% at the surveillance audit (Figure 3). Moreover, laboratories A and E both excelled in corrective action, increasing from 0% to 50% and from 8% and 67%, respectively, whilst laboratories C and D decreased from 8% to 0% and from 63% to 25%, respectively. When excluding internal audit and corrective action, the other QSEs for laboratory A and E improved by a median of 48 percentage points from exit to surveillance audit, whilst those from C and D improved by 15 percentage points. No other QSEs had such consistent results.

Ten nonconformities were common to all five laboratories: lack of critical procedures, lack of or incomplete management review records, incomplete personnel files, lack of equipment or method validation, lack of equipment calibration records, deficient internal audit, inconsistent internal quality control monitoring, unacceptable proficiency testing results, ineffective corrective action and deficient quality indicator monitoring (Table 1). At the exit audits, laboratories A and E had the most nonconformities. However, these laboratories developed corrective action plans that covered most of the nonconformities, so that by the surveillance audit their nonconformities were reduced by more than half. Laboratories C and D, on the other hand, reduced their nonconformities by only 12% – 40% at the surveillance audit, with many recurring issues.

**TABLE 1:** Number of nonconformities at exit and surveillance audits and implementation of the corrective action plan by the laboratory.

Laboratory	Number of nonconformities at exit audit	Number of nonconformities at surveillance audit	Coverage of nonconformities in corrective action plan (%)	Number of recurring^*^ nonconformities at surveillance audit^**^	
				*n*	%
Laboratory A	100	31	69	20	65
Laboratory B	23	19	17	12	63
Laboratory C	87	52	40	38	73
Laboratory D	60	53	12	30	56
Laboratory E	88	36	59	24	66

* Any nonconformity identified at both exit and surveillance audits in the same laboratory was classified as a recurring nonconformity.

** Common nonconformities (*N* = 10) included lack of critical procedures, lack of or incomplete management review records, incomplete personnel files, lack of equipment or method validation, lack of equipment calibration records, deficient internal audit, inconsistent internal quality control monitoring, unacceptable proficiency testing results, ineffective corrective action and deficient quality indicator monitoring.

## Discussion

All five laboratories in this study made notable improvements from exit to surveillance audit, displaying sustained and even increased efforts after completion of the SLMTA programme. Whilst laboratory B outperformed the other laboratories both at its exit and surveillance audits, this may in part reflect the smaller size, lower workload and less complicated management system of this small private research laboratory compared with the four hospital laboratories. Laboratories C and D reached one star at their surveillance audits, recording more modest improvements than laboratories A and E, which reached three stars despite having a higher number of tests per technician. Overall, results suggest that intensive mentorship provided post-SLMTA may be helpful with regard to accelerating quality improvement.

In an attempt to identify the factors associated with the relatively greater success of laboratories A and E, we examined closely their improvements by QSE. For the most part, all of the laboratories tended to struggle in the same areas (primarily internal audit, corrective action, and occurrence/management and process improvement) and excelled in the same areas (organisation and personnel, facilities and safety, and purchasing and inventory). However, laboratories A and E improved substantially in the internal audit QSE, increasing their scores from 0% to 60% in this area by the surveillance audit; laboratories C and D did not. They also improved well in corrective action, whilst the scores for this QSE decreased in laboratories C and D. Furthermore, improvements to these scores alone do not account for the greater overall improvements of laboratories A and E, as their scores in other areas also improved substantially more than laboratories C and D (48% versus 15%). The QSE ‘internal audit’ involves checking whether internal audits have been conducted by trained auditors and examining the corrective actions carried out to rectify the audit findings. ISO 15189 mandates that internal audits be conducted at least annually.^16^ Conducting internal audits can help a laboratory stay focused and provides concrete information with which to better understand its own areas of weakness and make decisions for improvement.^17^ The American Association for Laboratory Accreditation suggests that internal audits are critical because they allow laboratories to identify the root and potential causes of problems that need to be eliminated so that the problems are prevented from occurring the first time or recurring after correction. Subsequently, corrective or preventative action is taken to address a problem and eliminate the root cause of the problem.^18^ Thus, in laboratories A and E, internal audits and corrective actions may have acted as catalysts, facilitating improvement in other areas. In addition, discussion of internal audit results within the laboratory may have helped to develop an overall quality culture, not only amongst laboratory staff but also amongst hospital management, as has been identified elsewhere as a key factor in SLMTA success.^19^ Whilst this hypothesis is generated using a very small number of laboratories and various findings have been reported in other small studies,^20,21,22^ our results suggest that further evaluation of the benefits of conducting regular internal audits followed by corrective action is warranted.

The 12 QSEs can be divided into three quality stages: resource management (pre-analytical), process management (analytical) and improvement management (post-analytical).^23^ The three QSEs with the lowest scores for these laboratories at exit audit (internal audit, corrective action, and occurrence management and process improvement) are all part of the improvement management stage. Datema et al.^24^ point out that this stage is given the lowest scoring weight of the three quality stages in the SLIPTA system (16% of the total score, compared with 48% for resource management and 36% for process management in the original scoring structure; and 20%, 48% and 33%, respectively, in the current scoring structure).^15^ They argue that ‘this stage is of prime importance for sustaining the continuous improvement cycle’^24^ and that by awarding it fewer points, laboratories may be ‘less stimulated to invest effort in improvement management’.^24^

Overall, data on nonconformities obtained from the exit and surveillance audit reports showed that the actions taken to address deficiencies were often inadequate and, in most instances, root causes were not established and eliminated. The completeness and implementation of the corrective action work plan varied between laboratories. Laboratories A and E developed focused corrective action work plans based on the findings of the exit audit and implemented them thoroughly. In contrast, laboratories C and D made less progress in implementing corrective action plans. Of note is that, despite attaining four stars, laboratory B had 19 nonconformities at the surveillance audit, including 12 recurring nonconformities from the exit audit. It may be that this laboratory had already covered the easier parts of the checklist during SLMTA implementation and the remaining weak areas, such as occurrence management and internal audit, were hardest to implement and sustain post-SLMTA. Recurring nonconformities are graded as major events and could potentially lead to a serious breakdown of the QMS if not addressed in a timely fashion. Thus, even laboratories with high SLIPTA scores may still have major nonconformities that could pose a threat to sustainability of the QMS and prevent accreditation.

### Limitations

The circumstances in each country and in every laboratory within a country are unique, each facing multiple interconnected challenges. Whilst our analysis of five laboratories identified some interesting findings, it will be critical to expand this analysis using data from a large number of laboratories in multiple settings. This will give a better picture of performance trends across the 12 QSEs both during and after SLMTA implementation, as well as allowing for generalisation of the important issues facing laboratories today and the critical challenges impeding success and sustainability. Our study was limited by the lack of comparable baseline data from which to evaluate the common issues that existed before SLMTA implementation, as baseline data were excluded in order to minimise variability in auditor scoring. Finally, additional surveillance audits will be needed for the assessment of long-term sustainability of programme results.

### Conclusion

This analysis reveals common gaps in laboratory QSEs that can be addressed during and after SLMTA implementation. In particular, internal audits and subsequent corrective actions may play key roles in catalysing improvements in other areas, although a more systematic global evaluation is needed in order to generalise common problems and determine the best practices to address them.
